# Empowering Self-Efficacy by Using Patient Empowerment among Chronic Obstructive Pulmonary Disease: Pre–Post-Test Study

**DOI:** 10.3390/healthcare11030430

**Published:** 2023-02-02

**Authors:** Rasha Elsayed Ahmed, Izzeddin A. Bdair, Khalid AL-Mugheed, Shadia Hamoud Alshahrani, Mesheil M. Alalyani, Ramasubbamma Ramaiah, Seham I. Abdelrahman, Sanaa Ahmed Mahmoud, Mervat Moustafa Arrab

**Affiliations:** 1 Department of Medical-Surgical Nursing, College of Nursing, Tanta University, Tanta 31527, Egypt; 2 College of Nursing, King Khalid University, Khamis Mushait 61421, Saudi Arabia; 3 Department of Nursing, Al-Ghad International College for Applied Medical Sciences, Riyadh 23344, Saudi Arabia; 4 College of Nursing, Riyadh Elm University, Riyadh 11681, Saudi Arabia; 5 Department of Community Health Nursing, College of Nursing, Zagazig University, Zagazig 44519, Egypt; 6 Department of Pediatric Nursing, College of Nursing, Cairo University, Cairo 12613, Egypt; 7 Department of Family and Community Health Nursing, Faculty of Nursing, Menoufia University, Shibin El Kom 32511, Egypt

**Keywords:** chronic obstructive pulmonary disease, patient empowerment, self-efficacy

## Abstract

Patient empowerment is increasingly acknowledged as a milestone of high-quality patient-centered care. This study was conducted using COPD Self-Efficacy Scale to determine the effectiveness of the patient empowerment intervention program among chronic obstructive pulmonary disease patients on self-efficacy. We employed an interventional design with a pre-test and post-test. Sixty COPD patients comprised the final sample of the study. The current study revealed significant improvement in overall self-efficacy factors among most participants. Statistically significant positive correlations were found between the total self-efficacy post-empower intervention model scores concerning age, sex, work, educational level, and marital status. The study’s findings revealed that the patient empowerment intervention program positively affected COPD patients’ self-efficacy.

## 1. Introduction

Chronic Obstructive Pulmonary Disease (COPD) is a widespread and global health issue that affected around 251 million people in 2015 [1]. COPD is a chronic lung disease that prevents sufficient airflow in the respiratory tract and creates difficulties for sufferers [2]. Diminished lung function, quality of life, increased acute exacerbation, and shortness of breath characterize COP, and among others, symptoms include purulent sputum and alterations in the level of consciousness [3,4]. The prevalence of the disease in the adult population ranges from 6% to 10% globally, and approximately 3.2 million people die yearly from COPD [5]. The annual cost of COPD is estimated between US$6700 and US$13,400 per patient based on the cost of the medications used and associated medical problems [6,7]. COPD is expected to be the third leading cause of death worldwide by 2030 [1].

Chronic obstructive pulmonary patients face several negative psychological and physical experiences that impact their quality of life, such as sleep problems, reduced exercise capacity, fatigue, loss of appetite, and fear of death [8]. The quality of life of patients with COPD is impaired due to the inability to perform daily activities, prolonged hospitalization, and economic and social losses [9]. In addition, patients may feel threatened due to social, mental, and physical losses or changes, leading to challenges in the meaning of their lives [10,11,12,13]. Hence, these COPD-related negativities affect self-efficacy agency and decrease patient empowerment.

Patient empowerment interventions are a process by which patients earn greater monitoring power over decisions and actions impacting their health [14]. Patient empowerment is increasingly acknowledged as a milestone of high-quality patient-centered care [15]. Patient empowerment interventions ensure responsibility for the patients and get them involved in improving their health through participation and disease management [16]. Patient empowerment interventions are directed toward increasing knowledge and awareness, improving patients’ self-efficacy regarding self-control symptoms, supporting adopting preventive behaviors, and maintaining body functions [17].

Several studies have revealed that better patient empowerment is associated with positive health outcomes, e.g., self-management, self-efficacy, cost-effectiveness, and quality of life [18,19]. A systematic review of self-management interventions in COPD patient’s studies revealed decreased hospitalization rates, enhanced quality of life, and reduced dyspnea [20]. Using a COPD self-efficacy scale in a quasi-experimental study conducted among 60 COPD patients in Taiwan, Liou et al., found that a self-management program significantly improved patent self-efficacy [21].

Self-efficacy is an individual’s belief in his/her capacity to plan and conduct a path of action [22]. Self-efficacy plays a significant role in goal-directed behavior and keeping motivation and has been found to be a significant indicator of adherence to health behavior in medication usage, nutrition, and physical activity [23]. Self-efficacy can be general or specific [24]. General self-efficacy points to confidence in action management by a wide range of conditions, whereas specific self-efficacy points to confidence in action management relevant to a certain situation or behavior [22].

Self-efficacy has become broadly recognized as a promising approach for decreasing the COPD burden by assisting patients in positively changing their behaviors, improving accountability, and developing skills to manage their condition better [25]. Self-efficacy practices among COPD patients include stopping smoking, controlling and detecting symptoms, managing stress and psychological problems, and modifying lifestyle [26]. Improving self-efficacy practices could decrease hospital admissions, improve quality of life, and boost the empowerment of COPD patients.

Several studies have noticed that many COPD patients showed inadequate knowledge about the disease, its symptoms, and lifestyle [27,28]. According to the WHO, the main goal of the health education program is to assist patients in obtaining the necessary information to manage and adapt their lives to chronic diseases optimally [1]. Health education programs and non-pharmacologic interventions have been recommended in global COPD guidelines to enhance self-efficacy practice and boost patient knowledge [20,29].

Recently, research has focused on delivering COPD care via e-health platforms to provide a boost in COPD care capacity and healthcare access [30,31]. Systematic reviews found that using e-health use among COPD patients enhances collaboration between healthcare providers and patients and improved patient knowledge and skills [20]. Moreover, it facilitates regular monitoring clinical data of patients. A recent study collecting from 108 COPD patients found that e-health platforms were effective in delivering remote care for COPD patients at home. [32]. Therefore, e-health monitoring solutions can be considered as an alternative to traditional healthcare information and also improve quality care for patients [33,34]. In this regard, this is the first study conducted in Egypt to determine the effectiveness of a patient empowerment intervention program among chronic obstructive pulmonary disease patients on their self-efficacy.

Research hypotheses:

**Hypothesis 1 (H1).**  *Patients who complete the patient empowerment program will exhibit significant improvements in overall self-efficacy factors.*

**Hypothesis 2 (H2).**  *Are there any relationships between socio-demographic characteristics and self-efficacy factors?*

## 2. Materials and Methods

### 2.1. Design, Setting, and Sample

An interventional design with a pre-test and post-test was used. A convenience sample was drawn from a hospital database of outpatient pulmonary clinics and the inpatient ward of Tanta Main University Hospital in Tanta city in Egypt. The eligible criteria were inpatient wards and outpatient clinics for COPD patients using the Global Initiative for COPD (GOLD) guidelines and patients who had a forced expiratory volume of 50% or above in one second [35]. A total of 60 of 75 patients comprised the study’s final sample, while 15 patients chose not to participate.

### 2.2. COPD Self-Efficacy Scale

The study instrument had two sections. The first section included patients’ sociodemographic data, which include age, sex, marital status, education, occupation, family income, insurance, weight, height, and body mass index, as well as patient’s clinical profile data, including past medical history, causes of disease, duration, and stages of the disease. The second section used the COPD Self-Efficacy Scale (CSE), which Emme et al., (2012) developed to measure self-efficacy associated with COPD [28].

The scale includes 13 items with three factors. The first factor includes five items related to physical strains, such as going upstairs too fast, lifting heavy, hurrying or rushing around, and using poorly ventilated rooms during exercise. The second factor was weather/environment, with five items focusing on cigarette smoke, getting an infection, weather conditions, and air humidity. The third factor included three items related to proper diet, breathing, and overeating.

The scale uses a 5-point Likert-type scale, ranging from 5 (very confident) to 1 (no confidence). The overall score can range from (1–65), with a higher score indicating a greater level of self-efficacy, categorized as low (<60%), moderate (60% ≤ 80%), and high (≥80%). The scale was translated by professional language translators into the Arabic–Egypt national mother language. Three expert panels assessed tool validity, and a pilot study was performed with five patients. The Cronbach’s alpha of three factors were as follows: physical strains 0.87, weather/environment 0.77, and behavioral risk factor 0.81.

### 2.3. Patient Empowering Educational Manual

The patient-empowering intervention included an educational manual focused on instructional modules such as lifestyle modifications, dietary and physical guidance, awareness of COPD, drug prescription compliance, and self-management based on different guidelines such as Saudi Thoracic Society, 2014 [36], and Canadian Lung Association, 2022 [37]. The content of educational modules was readable at the elementary level and the Arabic–Egypt national mother languages were the main language. Three expert panels drawn from thoracic physicians, nurse educators and respiratory nurses, and dieticians assessed the content validity of the educational manuals. These manuals included many graphics besides each item to promote understanding and prevent misunderstanding. The content validity index of the educational manual was 0.83.

### 2.4. Data Collection

Before the data collection, ethical approval was received from the ethical review committee at Tanta University Nursing College with reference number (95-9-22). The researcher explained the aim of the study and study phases to the participants. They were informed that participation was voluntary, their data would keep at a high privacy level, and they had the right to drop out of the study at any time. After that, written consent was obtained from those who agreed to join the study.

The patient-empowering study comprised three phases: pre-intervention, intervention, and post-intervention. Data were collected from June to August 2019. Before the intervention phase, a self-efficacy survey was administered. The patients were taken to a quiet room in the hospital, and each participant received a self-efficacy survey and was asked to fill in the survey. They were returned within 15–25 min.

The intervention phase included several education methods such as face-to-face, problem-solving therapy, and e-health consultations such as videos, text messages, and follow-up calls. Within one week of sequential days, face-to-face sessions were conducted, ranging from approximately 35 to 45 min for each session. Face-to-face sessions focused on managing the disease’s signs and symptoms, controlling risk factors and threats, controlling functional disabilities, improving physical activity and breathing exercises, medical management, nutrition, and lifestyle modifications. The power-point presentation and booklets were used as teaching methods in face-to-face sessions with 16- to 40-point font sizes, and booklets included visual materials to increase readability.

The problem-solving therapy is one of innovation education [38,39]. In this study, it was performed by dividing the patients into groups; each group included six patients to facilitate their empowerment process based on emotion-oriented coping and problem-oriented. Through instructional videos, patients learned how to use inhalation devices and practice breathing and relaxation exercises. The length of the videos was 4–7 min. The researchers asked each patient to practice and repeat the video’s demonstration to become self-sufficient or empowered to the extent that he/she could do it without the presence of the researchers. E-health consultations, follow-up calls and text messages facilitated their continuous compliance with the intervention. The follow-up calls and text messages were performed by two researchers individually per patient with 10–20 min weekly chats over three months, using video consultations through Facebook messenger, WhatsApp, Zoom, and Skype. All follow-up consultations calls were recorded. After three months, the post-intervention phase was performed using the COPD Self-Efficacy Scale based on appointments of clinic visits in the selected hospital.

### 2.5. Data Analysis

SPSS software version 20 for Windows was used for the statistical analysis [40]. Descriptive statistics were conducted to describe the sample. The difference in the means between- and post-test scores in each group was conducted using paired *t*-tests. The Chi-squared test was used to evaluate differences in sociodemographic and self-efficacy factors. Multiple linear regression analysis was used to evaluate relationships between socio-demographic characteristics and self-efficacy factors. Statistical significance was set at *p* < 0.05.

## 3. Results

Table 1 shows that the age of the patients ranged from 35–55 years, with a mean age of 48.13 ± 5.18 years. About two-thirds (63.3%) of the studied subjects were male, and more than three-quarters (76.7%) were married. More than half (63.3%) of the patients had secondary education, and two-thirds (60%) were working. Nearly three-quarters (73.3%) of the participants had enough family income, and less than half (43.3%) had insurance.

Table 2 shows that nearly half (43.3%) of the study participants recorded that the cause of their disease was due to hereditary factors. About three-quarters (73.3%) of them reported that the duration of their disease was more than 10 years, more than one-third (36.6%) of the patients were exposed to three chest crises/year, and more than one-half (58.3%) of them were classified as having emphysema. Two-thirds (66.7%) of the patients were not smokers, and about three-quarters (73.3%) had no barrel chest. While about two-thirds (60%) of the studied patients had a cough, nearly three-quarters (73.3%) had shortness of breath. About one-fourth (26%) had a history of respiratory disorders.

The results revealed that 40% of patients had a mild stage of COPD, with FEV1 ≥80% predicted. Slightly more than one-third of patients (36.7%) experienced a moderate stage of COPD disease with (50% ≤ FEV1 < 80% predicted) airflow limitation and appeared with symptoms (cough, sputum production, dyspnea). Table 3 displays patients’ classification according to COPD stages.

Table 4 shows that overall self-efficacy factors showed a higher mean at the post-test than at the pre-test, followed by statistically significant improvement after the patient-empowering intervention (Pre-intervention = 5.7 ± 1.6, Post-intervention = 8.3 ± 2.1) (*p* < 0.001). There were also statistically significant improvements in the post-test regarding all self-efficacy factors.

There were significant differences between sociodemographic data concerning gender, marital status, and level of education, occupation, and total score of self-efficacy empowerment, *p* < 0.05. On the other hand, there were no significant differences between age groups or the total score of self-efficacy empowerment (*p =* 0.07). Table 5.

In Table 6. There was a statistically significant positive correlation between sociodemographic characteristics among the studied patients concerning gender, marital status, level of education, occupation, and total score of self-efficacy post empowerment, where *p* < 0.00.

## 4. Discussion

Patient empowerment is an ongoing process in which motivation, knowledge, and the ability to control their disease are built [41,42]. Improving self-efficacy of COPD patients is essential determinants of healthcare management outcomes and nursing interventions [43]. Thus, the current study aimed to determine the effect of the empowerment model on the knowledge and self-efficacy of COPD patients.

Results showed that this patient empowerment program provided effective interventions to enhance the self-efficacy of patients with COPD. The current study revealed significant improvements in overall self-efficacy factors among most participants. This result may be due to the positive effect of the empowerment intervention as the provision of patient support for problem-solving regarding maintaining functional ability, symptom control, and providing confidence relating to environmental changes and behavioral risk factors. This result aligned with other findings that comprehensive interventions significantly improve COPD patients’ self-management practices [29]. Rayyani et al., (2014) stated that empowerment, based on patients’ educational needs, promotes attitudes and behavioral signs toward their disease [44].

Using phone calls and text messaging is one component of the education program intervention to help patients improve their self-care and provide counseling [45,46]. A systematic review found that long-term health directions offered to patients via telephone significantly enhanced health status, health behavior, and self-efficacy [47]. Our findings also found that e-health interventions, including phone calls and short message services, improved the self-efficacy of patients with COPD. This approach was cost-effective, and patients were reachable, given the popularity of the telephone [48].

Regarding the patient clinical data, the current study reported that nearly one-half of the studied patients reported that the cause of COPD was due to hereditary factors, and more than one-half of COPD patients were diagnosed with emphysema; nearly two-thirds of them were non-smokers but had shortness of breath and cough, whereas about three-quarters of them did not have a barrel chest. This result aligned with other studies reporting that in this era, smoking was rare, but emphysema may occur in non-smokers, particularly with a familial predisposition or from environmental-provoking factors [19,49]. However, the current study’s findings did not align with Leiva-Fernández et al., (2014), who said that tobacco was a significant risk factor and an important cause in the initial diagnosis of COPD [50].

As regards the distribution of studied patients according to past medical history, the study findings revealed that about one-half of the studied patients had a history of a respiratory disorder. This may be because about one-half of the patients have hereditary causes of respiratory diseases. This result also agreed with a study that reported the common underlying disease was diabetes mellitus and hypertension, followed by cardiovascular and respiratory disorders [51].

However, the result of the current study was contradictory to several other studies. For example, a National Heart, Lung, and Blood Institute study stated that COPD most often occurs in patients with a smoking history [52]. In the United States, cigarette smoke is the leading cause, and pipes, cigars, and other types of tobacco smoke can also cause COPD. Ebrahim found that the cause of COPD was usually long-term exposure to irritant materials that damage the airways and lungs [53]. These contradictory results may be because of the comparatively smaller sample size, which warrants a longitudinal study with larger samples.

In the current study, there was a statistically significant positive correlation between sociodemographic characteristics among the studied patients concerning gender, marital status, level of education, occupation, and their total score of self-efficacy post empowerment *p* < 0.05. The results indicated that married male patients who can read, write and work had a good knowledge level and self-efficacy post-empowerment score, but there was no significant relation to age. This finding agreed with Berns, who reported no significant difference between the patient’s age and their total self-efficacy score [54]. This finding of the current study may suggest that the working males who could read and write were more interested in knowing about their disease and understood their role in preventing complications and coping with a new lifestyle according to the disease regardless of their age.

Wong et al., found a negative correlation between patients’ age and their self-care self-efficacy [14]. Salamah et al. [55] and Barham et al. [56] ensured that confidence levels according to age indicated that the oldest male had lower confidence than the youngest patient in terms of Cardiac Self-Efficacy. Tawalbeh et al. emphasized that systematic education, including a combination of verbal information and a booklet, could improve patients’ knowledge and problem-solving ability [57]. However, Effective clinical decision-making offered by nurses allows patients’ needs to be met, improving clinical outcomes and self-efficacy [58,59,60].

There are limitations of the study that should be addressed. The main limitations in our study was its low sample size and being conducted in one university hospital, which will be difficult to generalize. Further studies including larger populations can be suggested with different study design method.

## 5. Conclusions

In light of the current study’s findings, the conclusion can be reached that the empowerment intervention improved COPD patients’ self-efficacy. There was a significant positive correlation between sociodemographic characteristics regarding gender, marital status, level of education, occupation, and total score of self-efficacy post-empowerment. However, further studies are needed to emphasize the effect of the empowerment model on patients and caregivers by applying all dimensions of the empowerment model for patients with COPD. Continuing educational guidelines should be established for nurses who work with COPD patients to identify patients’ needs and assess their confidence level to improve patient self-care, self-efficacy, and problem-solving and decrease the burden level of disease.

## Figures and Tables

**Table 1:** Distribution of patients’ socio-demographic characteristics (n = 60).

Socio-Demographic Characteristics	Patients Sample	
	No	%
Age (year) Mean ± SD 48.13 ± 5.18		
30 > 40	2	3.3
41≥50	28	46.7
51 above	30	50.0
Gender		
Male	38	63.3
Female	22	36.7
Marital Status		
Married	46	76.7
Single	8	13.3
Widow	4	6.7
Divorced	2	3.3
Level of education		
Primary education	6	10.0
Secondary education	38	63.3
University education	10	16.7
Postgraduate education	6	10.0
Occupation		
Work	36	60.0
Do not work	18	30.0
Retired	6	10.0
Family income		
Enough	44	73.3
Not enough	16	26.7
Insurance		
Yes	26	43.3
No	34	56.7

**Table 2:** Distribution of the studied patients according to their clinical data (n = 60).

Clinical Data	No %	
Causes of COPD		
Hereditary causes	26	43.3
Acquired causes	8	13.3
Air pollution	12	20.0
Smoking	12	20.0
Others	2	3.3
Duration of disease		
<5 years	2	3.3
5–10 years	14	23.3
>10 years	44	73.3
How many chest crises do you have/ year?		
One	18	30.0
Two	18	30.0
Three	22	36.7
More than three	2	3.3
main forms of COPD		
Emphysema	35	58.3
Chronic bronchitis	25	41.7
Smoking		
No	40	66.7
Yes	20	33.3
Barrel chest		
No	44	73.3
Yes	16	26.7
Cough		
No	24	40.0
Yes	36	60.0
Shortness of breath		
No	16	26.7
Yes	44	73.3
Past Medical History	4	7.0
Heart Disease	2	3.0
Hypertension Disease	2	3.0
Peptic Ulcer	6	10.0
Diabetes	2	3.0
Renal Disease	26	44.0
Respiratory Disorders	4	7.0
Diabetic, Respiratory & Hypertension (more than one answer)		
No history of any illness	14	23.0

**Table 3:** Distribution of studied patients according to their COPD stages. (N = 60).

Classification of COPD Stages				
Stage	FEV1/FVC Ratio	Symptoms	N.	%
(0) at risk *	Normal spirometry	No have symptoms	0	0
(1) Mild	FEV1 ≥80% predicted	May have symptoms	24	40.0
(2) Moderate	50% ≤ FEV1 < 80% predicted	May have chronic symptoms	22	36.7
(3) Severe	30% ≤ FEV1 < 50% predicted	May have chronic symptoms	8	13.3
(4) Very severe	FEV1 <30% predicted.	Severe chronic symptoms	6	10.0

* COPD indicates chronic obstructive pulmonary disease; FEV1 forced expiratory volume in 1 s; FVC forced vital capacity. Symptoms (cough, sputum production, dyspnea).

**Table 4:** Comparison between categories and scores of self-efficacy pre- and post-empowerment (N = 60).

Categories of Self-Efficacy	Number of Items	Pre	Post	t	*p*
		Mean	(SD)	Mean (SD)	
Physical exertion factor	5	2.3 ± 1.2	3.9 ± 1.8	1.72	0.001 *
Weather/Environment factor	5	2.9 ± 1.7	4.1 ± 2.1	1.66	0.000 *
Behavior risk factor	3	1.3 ± 1.1	1.9 ± 1.3	0.58	0.001 *
Total	13	5.7 ± 1.6	8.3 ± 2.1	2.34	0.001 *

* Significant at *p* < 0.05.

**Table 5:** Relationship between sociodemographic data and the total self-efficacy empowerment score (N = 60).

Variable	Total Self-Efficacy Mean	*p* *
Age		
30–39	2.1 ± 1.5	0.07
40–49	4.2 ± 2.9	
≥50	4.4 ± 3.1	
Gender		
Male	6.2 ± 2.2	0.001 *
Female	4.4 ± 1.7	
Marital Status		
Married	8.1 ± 2.5	0.000 *
Single	5.5 ± 2.1	
Widow	3.8 ± 1.5	
Divorced	2.2 ± 1.1	
Level of education		
Primary	2.5 ± 1.4	0.001 *
Secondary	9.3 ± 3.7	
University	6.5 ± 2.6	
Postgraduate education	4.2 ± 1.9	
Occupation		
Work	7.7 ± 3.1	0.001 *
Do not work	4.8 ± 2.1	
Retired	2.3 ± 1.1	

* Significant at *p* < 0.05.

**Table 6:** Multiple linear regression analysis results: sociodemographic characteristics associated with total self-efficacy score. (N = 60).

Socio-Demographic Characteristics	Total Score of Self- Self-Efficacy (Post)		
	R	F	P
Age (year)	0.067	57.2	−0.216
Gender	0.789	22.1	(0.001 *)
Marital Status	0.641	45.6	(0.002 *)
Level of education	0.872	29.3	(0.000 *)
Occupation	0.624	33.1	(0.000 *)

* Significant or *p* < 0.0; Pearson Correlation: R.

## Data Availability

The data presented in this study are available on request from the corresponding author.
